# A molecular epidemiological study of rabies epizootics in kudu (*Tragelaphus strepsiceros*) in Namibia

**DOI:** 10.1186/1746-6148-2-2

**Published:** 2006-01-13

**Authors:** Karen Mansfield, Lorraine McElhinney, Otto Hübschle, Felix Mettler, Claude Sabeta, Louis H Nel, Anthony R Fooks

**Affiliations:** 1 Rabies and Wildlife Zoonoses Group, Veterinary Laboratories Agency (VLA, Weybridge), WHO Collaborating Centre for the Characterisation of Rabies and Rabies-Related Viruses, New Haw, Addlestone, Surrey, KT15 3NB, UK; 2 Virology Department, Central Veterinary Laboratory, Windhoek, Namibia; 3 Claude Sabeta Onderstepoort Veterinary Institute (OVI), Virology Department, Rabies Unit, Old Soutpan Road, P Bag X05, Onderstepoort 0110, Pretoria, South Africa; 4 Department of Microbiology and Plant Pathology, Faculty of Natural and Agricultural Sciences, Lynwood Road, University of Pretoria, 0001 Pretoria, South Africa

## Abstract

**Background:**

A panel of 37 rabies virus isolates were collected and studied, originating mainly from the northern and central regions of Namibia, between 1980 and 2003.

**Results:**

These virus isolates demonstrated a high degree of genetic similarity with respect to a 400 bp region of the nucleoprotein gene, with the virus isolates originating from kudu antelope (*n = 10*) sharing 97.2–100% similarity with jackal isolates, and 97–100% similarity with those isolated from domestic dogs. Phylogenetic analysis suggested that these viruses were all of the canid rabies biotype of southern Africa. The viruses from kudu were closely associated with jackal isolates (*n = 6*), bat-eared fox isolates (*n = 2*) and domestic dog isolates (*n = 2*) at the genetic level and identical at the amino acid level, irrespective of the year of isolation.

**Conclusion:**

These data suggest that jackal and kudu may form part of the same epidemiological cycle of rabies in Namibian wildlife, and might demonstrate the close-relationship between rabies virus strains that circulate within Namibia and those that circulate between Namibia and its neighbouring countries such as Botswana and South Africa.

## Background

**Figure 1:**
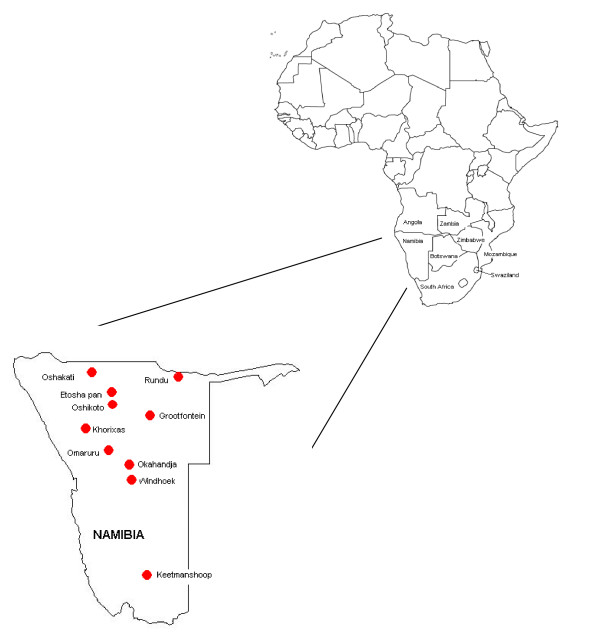
Map showing the location of Namibia within the African continent and the location of Namibian isolates.

The Republic of Namibia is a large country of 824268 km^2 ^situated on the south Atlantic (West) coast of Africa. It shares borders with South Africa in the south, Botswana and Zimbabwe in the east, and Angola and Zambia in the north (Figure 1 – refer to additional file 1). Namibia is sparsely populated, with the Namib desert stretching along the western coast and the Kalahari desert along the south-eastern border with Botswana. However, Namibia provides for an abundance of wildlife with its twenty-six parks and nature reserves, the most familiar of which is the Etosha National Park in the north of the country.

Classical rabies virus (RABV; genotype 1) has a single-stranded, negative-sense RNA genome, and is a member of the *Rhabdoviridae *family, within the genus *Lyssavirus*. In addition to RABV, the lyssaviruses circulating in Africa include Lagos bat virus (genotype 2), Mokola virus (genotype 3) and Duvenhage virus (genotype 4) [1]. Classical rabies viruses circulating in southern Africa can be further sub-divided into two distinct biotypes, canid and viverrid [2]. Monoclonal antibody typing has confirmed the existence of these two distinct groups, with the wildlife-associated mongoose (viverrid) biotype appearing more phylogenetically diverse [2-5]. The canid biotype infects carnivores of the family Canidae, primarily the domestic dog (*Canis familiaris*), jackal (*Canis mesomelas*, the black-backed jackal) and bat-eared foxes (*Otocyon megalotis*) [5]. The mongoose (viverrid) biotype principally infects the yellow mongoose (*Cynictis penicillata*) which maintains the virus, whereas in reality there is no evidence that true viverrids (such as civets and genets) are maintenance hosts of RABV [5]. Mongoose rabies is likely to have been endemic throughout southern Africa (including Namibia) prior to the advent of canine rabies. Although of importance over the vast area of southern Africa, mongoose rabies is less threatening to human and animal health and is consequently difficult to trace in history [5].

Rabies is a notifiable disease in Namibia [6]. Here, as early as 1887, a disease outbreak among dogs, cattle and other livestock was presumed to be rabies, given the disease symptoms [7,8]. Nevertheless, the first confirmed case dates back to 1906, to the coastal town of Swakopmund [8]. Rabies is particularly common in the northern parts of Namibia; the Ovambo, Kavango and the Caprivi (bordering on Angola and Zambia), where sporadic reports of rabies (unconfirmed) involving dogs, bovines and humans occurred throughout the latter half of the 1920s. These reports of rabies coincided with reports from the southern parts of neighbouring Angola [1,8]. For example, in Ovamboland, northern Namibia, a number of people died at a Mission Hospital in 1926, with hydrophobia and a history of having been bitten by rabid dogs [9]. By the end of World War II in the 1940s, a rabies epizootic ensued in Angola and Zambia (then Northern Rhodesia) and spread southward into Namibia and Botswana. It is probable that dogs were solely responsible initially, but jackals soon became an important reservoir and remains so at the present time [10]. Thus canine rabies entered Botswana and Namibia during the late 1940s from southern Angola/Zambia [6], and was later found to be phylogenetically different from the endemic mongoose (viverrid) strains previously circulating in these regions [3]. Rabies cycles in the black-backed jackal appeared soon after the introduction of dog rabies in Namibia [11]. Thereafter, with the involvement of jackals, the disease spread southwards, past the Etosha National Park to reach Outjo, then Otjiwarongo and later the capital Windhoek, by 1951 [12]. This canine epizootic continued to spread through Namibia and Botswana and into the northern Transvaal (Republic of South Africa) during the early 1950s, where it spilled over into jackals and bat-eared foxes (*Otocyon megalotis*) [2]. Canid rabies in southern Africa is thought to be derived from a single virus lineage that most probably originated from Europe and may have been introduced into West Africa by slave-trading Europeans, after 1500 AD. Phylogenetically, the viruses capable of maintaining prolonged and independent cycles of disease throughout canid host populations of sub-saharan Africa, particularly domestic dogs, jackals and bat-eared foxes, are strongly linked to the global 'cosmopolitan lineage' of rabies virus strains of European descent [13,14].

By the mid-1970s there was sporadic but endemic rabies throughout most of Namibia, generally with dog and human rabies in the more populous north, jackal and cattle rabies in the central ranching areas and sporadic canid or mongoose rabies in the arid sheep farming areas of the south [1,8].

The unusual occurrence of rabies in the kudu antelope (*Tragelaphus strepsiceros*) was first observed in 1975 near Windhoek. However an epizootic of rabies in these antelopes began to the north in Okahandja district in 1977 and was in the same year that rabies was first confirmed in two kudu in the Etosha National Park. The number of confirmed cases rose steadily throughout 1978–1979 [15], spreading westwards along the Swakop River and then north and south during 1978 [8]. The latter part of the epizootic (1983–1984) coincided with the first cases of lions contracting the disease in the Etosha National Park. It is thought that the lions became infected from hunting rabid kudu, as all four reports of rabid lions were from an area of high kudu population density in eastern Etosha [16]. This kudu epizootic peaked in 1980, but had eventually subsided by 1985 [8], by which time it caused an estimated loss of 30–50 000 antelope, or 20% of the population [1]. However, during 2002 there was another substantial outbreak in kudu, where an estimated 2500 animals on more than 81 farms in Namibia died [17]. This outbreak continued into 2003.

The kudu (*Tragelaphus strepsiceros*) is a wild ruminant with spiralling twisted horns, and is generally found in areas of broken rocky terrain where there is easy access to water [8]. In Namibia, the main food source for the kudu is the leaves of *Acacia *species, including *Acacia hereroensis *(Berg thorn), along with a variety of other plants in the savannah woodland [8]. They spend most of the year living in small herds of four to six animals [8] which eat and move together, and have close contact with each other through activities such as mutual grooming [15]. These groups will split up during the breeding season, but will come together again later. Contact between different social groups can occur at watering places, along with the possibility of contact with other species [15]. Farm fencing that is usually effective for cattle and gemsbok does not control the movement of kudu [15], as the kudu can easily jump a 1.2 m fence [8]. The social behaviour of kudu has contributed to the spread of rabies, and it is thought that mouth lesions from the browsing of thorn-bushes may have been a contributing factor due to the presence of RABV in saliva [15].

The kudu epizootics in Namibia have provided an example of non-bite transmission, with horizontal spread between kudus [18], and posed a threat to human health via the game breeding and hunting industries in Namibia. At the time of the 1977–1983 epizootic, kudu constituted more than 60% of the game farming trade, therefore rabies-infected kudu were a threat to both the consumer and hunter of game [15].

Phylogenetic analysis has been a useful tool in the study of rabies epidemiology [19], and will aid in establishing divergence in the RABV isolates being studied throughout the world. In this study, phylogenetic analysis of a partial region of the nucleoprotein gene was undertaken, on a panel of 60 sequences derived from rabies viruses from Namibia (n = 37 from our study; n = 3 from Genbank) and neighbouring countries. The Namibian panel included eleven sequences from kudu, isolated in 1980 (n = 1), 1987 (n = 1, obtained from Genbank) and 2003 (n = 9) at the time of the second kudu epizootic. The aim of this study was to provide an insight into the rabies situation in Namibia, in particular the complex interaction between urban and sylvatic cycles of RABV, and the maintenance of cycles in different species including kudu.

## Results

**Figure 2:**
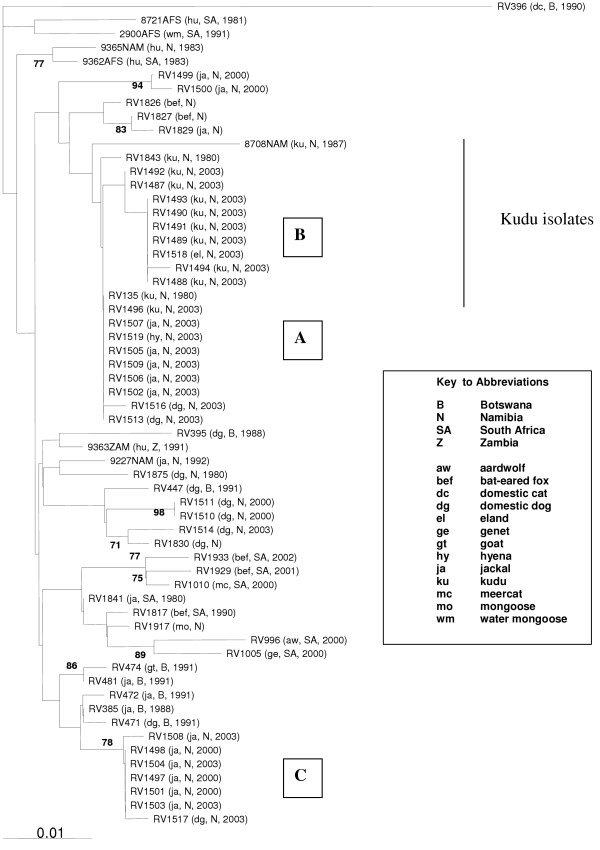
Phylogenetic relationship between 60 rabies viruses from Namibia and neighbouring countries in Southern Africa based on a 400 bp region of the nucleoprotein gene. A rabies virus isolated from a domestic cat in Botswana was used as an outgroup. Only bootstrap values exceeding 70% are shown.

A cohort of 40 Namibian RABV sequences, mainly isolated in the central to northern parts of the country and from a variety of species, but including 11 isolates from kudu antelope, were studied. Cluster analysis of the nucleotide sequence of a 400 bp region of the nucleoprotein gene demonstrated 22 unique sequences. Three of these sequences were obtained for multiple isolates and are represented collectively by groups A-C (Figure 2 – refer to additional file 2).

Strains from the most frequently recovered group (Group A) were geographically located in the northern districts of Grootfontein and Etosha, and contained one virus isolate from kudu, two from jackal and one from a dog from Grootfontein (RV1496, RV1502, RV1509, RV1513), along with three isolates from jackals and one hyena sample from the Etosha National Park (RV1505, RV1506, RV1507, RV1519). However, strains of group A were not restricted to this area. This group also included a single isolate (RV135), which was collected from a kudu in South Africa in 1980.

A second group of identical sequences (Group B) originated from more central regions of Namibia and comprised two isolates from kudu from the Okahandja district (RV1490, RV1493), two isolates from kudu and one from an eland from the Omaruru district (RV1489, RV1491, RV1518), and one isolate from a kudu from Windhoek (RV1488). The jackal group (Group C) contained three isolates from jackals around Windhoek (RV1501, RV1503, RV1504), one from a jackal in the Khorixas district (RV1498) and one from a jackal in the Okahandja district (RV1497). Also demonstrating 100% similarity with respect to this region of the N-gene were two virus isolates from kudu from the neighbouring regions of Windhoek and Okahandja (RV1487, RV1492). Finally, two virus isolates from dogs in the Oshikoto and Oshakati districts (RV1510, RV1511) were also found to share identical sequences. With the exception of RV135, sequences represented by multiple isolates were geographically localised (Figure 1 – refer to additional file 1). The remaining 17 unique sequences represented Namibian isolates collected from kudu, jackal, bat eared foxes, dogs, humans and a single mongoose. The Namibian isolates formed a large diffuse group representing a canid biotype and were closely associated with previously reported Africa 1b isolates [20] (Table 1). The mongoose isolate (RV1917), collected from Keetmanshoop in Southern Namibia, was more similar to bat-eared fox isolates from South Africa than to the other Namibian isolates or to the previously designated mongoose biotype isolate RV396 (Figure 2 – refer to additional file 2).

The phylogenetic relationships between the Namibian rabies virus isolates and those from neighbouring countries for the 400 bp region of the N-gene are shown with isolate RV396 included as an outgroup (Figure 2 – refer to additional file 2). Urban and sylvatic Namibian isolates were not resolved into two clear groups in the tree. Indeed dog and jackal isolates were distributed throughout the Namibian group. A discrete cluster of domestic dog isolates (RV1830, RV1514, RV447, RV395, RV1875, RV1510 and RV1511) may represent an urban cycle, however, this group does not have full bootstrap support and includes a single jackal isolate (9227NAM).

The Namibian isolates are seen to cluster in two groups, however, the resolution of these groups is not supported by bootstrap analysis. The upper group comprised sequences from all of the kudu, 8 jackals, 2 dogs, 2 bat-eared foxes, an eland and a hyena. With the exception of the kudu isolate from South Africa (RV135), all of these isolates originated from Namibia. With the exception of RV1500 and RV1499, all of the isolates in the upper cluster had identical deduced amino acid sequences in the 400 bp region.

Unlike all other species analysed in this study, all of the virus isolates from kudu clustered together (Figure 2 – refer to additional file 2). Isolates from species such as jackals and domestic dogs were dispersed throughout the tree. Five of the kudu isolates from Central Namibia (Group B) shared 100% sequence similarity with an isolate from eland antelope (RV1518) from Omaruru suggesting that the kudu and eland hosts of this viral strain originate from a similar geographical area in Namibia, and are involved in the same epidemiological cycle (Figure 1 – refer to additional file 1). Kudu isolates from the most recent epizootic are closely related to two earlier (1980) isolates obtained from kudu near Windhoek, Namibia (RV1843) and South Africa (RV135). Grouping closely (but more distantly) was a kudu rabies isolate from 1987 (8708NAM). The lower group comprises several clusters representing various isolates from Botswana and South Africa along with more discrete clusters of jackals and domestic dogs from Namibia. Of the five domestic dog viruses within this group, suggestive of an urban cycle, four were isolated from dogs in northernmost parts of Namibia (RV1510, RV1511, RV1514, RV1830), whilst the fifth was identified in a dog in Maun, in Botswana (RV447). These cases are clearly related and probably indicate a dissemination event related to the transport of companion animals. This group also contains another dog from Windhoek in Namibia (RV1875), and a dog from Mochudi in neighbouring Botswana (RV395). Interestingly, the six isolates from Namibian jackals (Group C, RV1508) and one from a Namibian dog (RV1517), are closely related to a cohort of isolates from Botswana from jackals (RV385, RV472, RV481), domestic goats (RV474, RV475), a dog (RV471) and a cow (RV386). These data demonstrate the dispersion of closely-related canid viruses within Namibia and neighbouring Botswana.

A jackal isolate (RV1841) is the most similar of the South Africa Group to the Namibian and Botswanan canid isolates demonstrating again the close relationship between isolates from neighbouring countries due to the movement of such species. The mongoose species responsible for isolate RV1917 has not been identified but it is most likely to have been a yellow mongoose, which is commonly found in the Southern Namibian region of Keetmanshoop. The spillover of canid biotype into mongoose hosts is also demonstrated by the inclusion of the South African water mongoose isolate (2900AFS) (Figure 2 – refer to additional file 2).

Dendrograms generated for the partial G-gene (nucleotide co-ordinates 3375–3974) and N-gene analyses were congruent (data not shown). Furthermore, phylogenetic analysis of the concatenated sequence (1000 bp) yielded no significant improvements in terms of either the bootstrap values or topography (data not shown). Phylogenetic analyses of the dataset using either the maximum likelihood (DNAML) or parsimony (DNAPARS) programmes within the PHYLIP package did not improve the resolution of isolates or demonstrate significant differences in topography or bootstrap support (data not shown) which lends support to the co-existence of closely related urban and sylvatic rabies cycles in Southern Africa.

## Discussion

The molecular sequence and phylogenetic analysis of the Namibian rabies virus isolates described here, has demonstrated a high degree of similarity between isolates originating from different species, emphasising clearly the ease of transfer of RABV throughout Namibian wildlife, and between wildlife and domesticated species.

The demonstration of two epidemiological cycles of RABV circulating in southern Africa is well documented. Within the canid biotype of RABV there appears to exist two epidemiological cycles [21]; an urban (canid) cycle within the domestic dog population, and a wildlife (or sylvatic) cycle which occurs among the jackal, kudu and bat-eared fox populations. Interestingly, the isolates from mongooses (*Viverridae*) have been shown to be phylogenetically distinct from the canid biotype [3]. Our data provides further evidence for the existence of these two cycles in Namibia. However, urban and sylvatic isolates were not fully resolved on our tree, which indicates that the wildlife and urban cycles of RABV existing in Namibia are closely-related. Indeed, our data suggests that canid isolates from Namibia, South Africa, Botswana and Zambia form a single diffuse group representing both urban and sylvatic cycles which interact with each other and may have a recent common ancestor. The propensity of certain strains of RABV to survive may be host-dependant, and it has been suggested that the evolution of viruses may reflect that of the host species [5]. This may lead to the suggestion that the kudu clade simply indicates a host switch or host range expansion unique to a particular lineage of RABV, as the passage of viruses within different host species may enhance the evolution of the viruses when they are being cycled among a number of different host species [5]. However, although the process of recombination can aid the generation and spread of advantageous genotypes (natural selection), it has previously been shown that recombination is unlikely in negative-sense RNA viruses such as RABV [22].

A number of clinical signs have been observed in kudu infected with RABV, the most common of which is loss of fear [23]. An infected kudu often appears completely oblivious to its surroundings; it loses its fear of both domestic animals and man, and has often been observed walking into houses and garages and even approaching vehicles. This may explain a likely route of transmission and infection of the two dogs with a sylvatic strain of RABV; the dogs may have bitten or licked the dead or dying kudu. A larger data set may determine the nature of the domestic dog isolates (RV1513 and RV1516) in what would otherwise be a cluster of sylvatic isolates. However, the addition of more viruses may provide further evidence of a common urban and sylvatic canine rabies cycle observed in previous studies [3,21]. Previous work has suggested that the cycles of RABV are maintained in the jackal population, and that dog cases result from fights with rabid jackals [6]. Although the jackal is not a domesticated species, when infected with RABV it can appear 'tame' and is commonly observed around watering places where it can attack cattle, domestic animals and humans [21]. It is clear that jackal and dog rabies cycles are closely linked to such an extent that the likelihood of jackals sustaining independent rabies cycles remains a controversial issue. These data confirm however, the interconnected nature of dog and jackal rabies cycles in southern Africa.

RABV clearly poses a threat to human and animal health in Namibia and throughout the African continent, as demonstrated by the similarity between viruses from neighbouring countries. The highest population density in Namibia is located in the north of the country, where there are many dogs and little wildlife. In this region, rabies is detected mainly in dogs, and poses a threat to human and bovine health [6]. According to OIE figures, and excluding kudu data, cases of animal rabies in Namibia have been increasing gradually, with 123 in 2000, 153 in 2001, 162 in 2002 and 214 reported cases in 2003 [17]. This increase in cases of canid rabies in the north of Namibia has continued to escalate in 2004 and 2005 and constitutes, at the present time, an increasingly serious problem of human and animal health in northern to central Namibia, for which urgent control actions are required to prevent human fatalities. During 2003, most of these cases were recorded in domestic dogs, cattle and wildlife. The majority of reported cases in Namibia are found in the central stock-ranching region, where human population density is low, and wildlife plentiful [6]. In this region of large commercial farms, most cases are found in cattle and the black-backed jackal, and appear to follow a seasonal pattern, with an increase in cases in all species from June to November. During these months, rainfall is low and animals have to increase their home range in the search for water and food [6].

During the years preceding the 1977–1983 rabies epizootic in kudu, above-average rainfall and abundant growth of vegetation made Namibia a very favourable environment for kudu and the population had greatly increased [8]. Furthermore, farmers had exterminated many large predators of the kudu because they also killed livestock, and there was little hunting of the kudu due to the preservation of the species for 'trophy-hunting' tourism. All of these factors contributed towards a large overpopulation of kudu in Namibia [8], which further assisted the spread of the epizootic. During the epizootic, severe drought conditions may have forced the kudu to move further north in the search for more plentiful supplies of food and water [8], which caused further dissemination of the disease.

Our analysis confirms that the RABV strains that infected the kudu population during both epizootics was of the canid biotype. This biotype however, was phylogenetically distinguishable from the group of viruses isolated from *Herpestidae *such as the yellow mongoose [3]. The earlier epizootic in kudu was most likely initiated by a jackal bite, as peaks of rabies incidence in kudu and cattle coincided with peaks in jackals, although the incidence in kudu appeared to far exceed that in jackals [8]. Furthermore, our results suggest that the jackal and kudu share the same epidemiological cycle of rabies in Namibian wildlife, and that the probable source of infection in the later 2002 epizootic was from the canid biotype most probably the black-backed jackal or perhaps the domestic dog, as genetic analysis grouped these species most closely with the kudu. Although the jackal is the most likely candidate, from a behavioural and ecological point of view, bat-eared foxes are considered an ideal vector for the transmission of RABV [24]. However, although our results show two bat-eared foxes from Etosha (RV1826, RV1827) also grouping with the kudu, they are not clustering as closely to the kudu as the dogs or jackals so are less likely to be the source of infection.

The high degree of similarity among the kudu isolates suggests that once RABV enters this species it spreads rapidly through the population with little interaction with other species. The kudu population then appears to maintain its own cycle of RABV infection. Epizootic observations at the time of the kudu outbreak suggest a horizontal spread of RABV throughout the species, particularly as the only other wild herbivore affected was the eland [23]. The number of confirmed cases, the rate of propagation and the size of the area affected suggest that it would have been impossible for such a large number of cases to have been caused by other species such as the jackal or mongoose. The outbreak of RABV in kudu was an epidemiological rare event, and any reasons as to why rabies was maintained within the kudu population are purely speculative. Since the kudu are not a naturally aggressive species, this would suggest that the spread of virus throughout the population was by non-bite transmission, and the horizontal spread of RABV from kudu to kudu via a non-bite route has previously been suggested [15]; indeed studies have shown that kudu are highly susceptible to oral and/or nasal infection, with infected kudu often having a high concentration of virus in their saliva [18]. Kudu commonly receive mouth lesions from grazing on the thorny acacia plants, which are the first trees to bud in spring. Grazing kudu can leave saliva deposits on the acacia plants, which may contain RABV if the animal is infected. The thorns of the acacias are inconspicuous, but can cause severe wounds which provide a prime route of entry for the virus [8]. Unlike other species, kudu are social-leaf eaters, often browsing the same plant together [25], and providing an easy mode of transmission. Subsequently, we postulate that this is also the most likely route of virus transmission from kudu to kudu during the latter 2002 epizootic in Namibia.

This theory is however, not exclusive. An alternative hypothesis for the non-bite route of transmission of RABV throughout the kudu population has been suggested. Kudu live in small herds which remain together, moving and feeding at the time and living in relatively close contact [8]. One of the most common symptoms of a rabies infection in kudu is the production of continuous copious amounts of saliva [8]. Mutual grooming is common in animals which exist in small close groups, so a kudu attempting to lick a dead or dying RABV-infected kudu could potentially come into contact with infected saliva, which is then transmitted to the healthy kudu through any thorn lesions in the mouth. It is likely that the transmission cycle could be maintained by a combination of these proposed hypothetical routes. In addition, with the initial introduction from a canine cycle into a kudu cycle, secondary kudus may also become infected by a victim animal through the licking of an open wound which has retained infected saliva obtained from attack by a rabid animal (such as a jackal). This theory is similar to that applied to mongooses which live in communal burrows and are thought to transmit RABV by the salivary gland route [5].

Our data suggest that the isolates studied show a high degree of genetic similarity between a range of species and geographical locations and demonstrates how closely-related the RABV strains circulating in Namibia are to those from neighbouring countries such as Botswana and South Africa.

## Conclusion

The epidemiological cycles (urban and sylvatic) of RABV circulating throughout the countries of southern Africa are complex, with a large number of vector species and the existence of at least two distinct biotypes of RABV (mongoose and canid). Different species appear to maintain their own cycles of infection, such as that by non-bite transmission seen in the kudu during the epizootics. Our data suggests that jackal and kudu may form part of the same epidemiological cycle of rabies in Namibian wildlife. The canid biotype is now highly established throughout southern Africa. This makes any control programme difficult to implement, due to the large variation in species and habitat. We believe that rabies is highly prevalent and widespread in southern Africa and that the disease poses a continual threat to a large variety of wildlife species, to livestock and to human health.

## Methods

### Viral isolates

A panel of thirty-seven viruses isolated from kudu (*Tragelaphus strepsiceros*), black-backed jackal (*Canis mesomelas*), domestic dog (*Canis familiaris*), eland (*Taurotragus oryx*), bat-eared fox (*Otocyon megalotis*), mongoose and hyena (*Crocuta crocuta*) between 1980 and 2003 were sequenced (Table 1). With the exception of the mongoose isolate (RV1917), the viruses were isolated from host species from a wide area in the northern and central regions of Namibia (Figure 1 – refer to additional file 1, Table 1). One kudu isolate was collected in Namibia, in 1980, during the first epizootic. Nine kudu isolates were obtained in 2003, at the time of the second epizootic.

Previously published RABV nucleotide sequences from Namibia (n = 3) and the neighbouring countries of Botswana, Zambia and South Africa were also included in the analysis (Table 1). A domestic cat isolate from Botswana (RV396), previously designated as belonging to the viverrid (mongoose) biotype was included as an outgroup (Table 1).

### Extraction of RNA

RNA was extracted using TRIzol^® ^(Invitrogen) following the manufacturer's instructions, and resuspended in HPLC purified water (Sigma Aldrich). RNA was then diluted to 1 μg/μl in HPLC water, and stored at -80C.

### RT-PCR and sequencing

Reverse transcription and polymerase chain reaction were performed as described previously [4] using the combination of primers JW12, JW6 DPL, JW6 M and JW6 E. PCR cycling was as described previously [26]. Positive reactions produced a 606 base pair (bp) fragment, corresponding to position 55 – 660 of the nucleoprotein gene.

PCR products were purified using the QIAquick™ PCR Purification Kit, and sequenced in both directions using either the Big Dye Kit (Applied Biosystems), or the Beckman QuickStart kit (Beckman-Coulter), with primers at 3.2 pmole/μl. Forward sequences were produced with primer JW12, whereas reverse sequences were obtained using either JW6 DPL or JW6 UNI (CARTTVGCRCACATYTTRTG).

### Phylogenetic analysis

Nucleotide sequences were edited to 400 bp using the DNAstar programme (DNAstar Inc. Madison, USA). Multiple sequence alignments were generated using the Clustal W programme [27] and the transition-transversion ratio (2.73) was estimated using the Puzzle programme (Version 4.0.2) [28]. The data set was analysed using the DNAdist (maximum likelihood parameter) and Neighbour Joining programmes within the PHYLIP package [29]. For comparison the dataset was also analysed using the DNAML and DNAPARS programmes within the PHYLIP package. Bootstrap re-sampling with 1000 replicates using the Seqboot, DNAdist and Neighbour programmes provided confidence limits for the constructed phylogenies, and the consensus tree was generated within the Consense programme. The phylogenetic tree was generated and bootstrap values were visualised using Treeview [30]. Bootstrap values are expressed as percentages.

Additional sequence analysis was performed with a reduced dataset, using a 600 bp region of the glycoprotein gene (nucleotide co-ordinates 3375 – 3974). Sequences from the glycoprotein gene were derived as previously described [19]. A 1000 base pair concatenated sequence comprising 400 bp of the nucleoprotein gene and a 600 bp region of the glycoprotein was also analysed.

## Competing interests

The authors' declare that they have no financial or personal relationships with other people or organisations that could inappropriately influence this research study. All authors have access to all data in the study and held final responsibility for the decision to submit for publication.

## Authors' contributions

KM performed data analysis and interpretation, RT-PCR and sequencing, phylogenetic tree construction, figure preparation and writing the manuscript. LMcE participated in phylogenetic tree construction, data interpretation and writing the manuscript. OH and FM co-ordinated the study with veterinarians in Namibia and supplied the panel of Namibian isolates for analysis. CS participated in data analysis and in supplying the panel of Namibian isolates and supplied archived Namibian isolates for analysis. LN participated in data interpretation and writing the manuscript. ARF conceived and co-ordinated the study with veterinarians in Namibia and participated in study design, data analysis, data interpretation and writing the manuscript. All authors read and approved the final manuscript.

## Figures and Tables

**Table 1:** Rabies viruses included in the phylogenetic analysis

**ID Number**	**Original ref**	**Country**	**Location**	**Species**	**Year**	**Genbank**
RV385	R12/9/88	Botswana	Ghanzi	Jackal	1988	AY330733
RV395	R11/8/88	Botswana	Mochudi	Dog	1988	AY330743
RV396	R13/12/90	Botswana	Lobatse	Domestic cat	1990	AY330744
RV447	R16/6/91	Botswana	Maun	Dog	1991	AY330752
RV471	R3/10/91	Botswana	Maun	Dog	1991	AY330753
RV472	R4/10/91	Botswana	Maun	Jackal	1991	AY330754
RV474	R7/9/91	Botswana	Palapye	Goat	1991	AY330756
RV481	R2/8/91	Botswana	Orapa	Jackal	1991	AY330761
RV1487	1380/05/03	Namibia	Windhoek	Kudu	2003	DQ194855
RV1488	1383/05/03	Namibia	Windhoek	Kudu	2003	DQ194856
RV1489	1418/05/03	Namibia	Omaruru	Kudu	2003	DQ194857
RV1490	1715/05/03	Namibia	Okahandja	Kudu	2003	DQ194858
RV1491	1784/06/03	Namibia	Omaruru	Kudu	2003	DQ194859
RV1492	1938/07/03	Namibia	Okahandja	Kudu	2003	DQ194860
RV1493	2161/07/03	Namibia	Okahandja	Kudu	2003	DQ194861
RV1494	2213/07/03	Namibia	Omaruru	Kudu	2003	DQ194862
RV1496	3258/10/03	Namibia	Grootfontein	Kudu	2003	DQ194863
RV1497	2295/09/00	Namibia	Okahandja	Jackal	2000	DQ194864
RV1498	2318/10/00	Namibia	Khorixas	Jackal	2000	DQ194865
RV1499	2363/10/00	Namibia	Etosha National Park	Jackal	2000	DQ194866
RV1500	2832/11/00	Namibia	Etosha National Park	Jackal	2000	DQ194867
RV1501	2847/11/00	Namibia	Windhoek	Jackal	2000	DQ194868
RV1502	1907/07/03	Namibia	Grootfontein	Jackal	2003	DQ194869
RV1503	2141/07/03	Namibia	Windhoek	Jackal	2003	DQ194870
RV1504	2259/07/03	Namibia	Windhoek	Jackal	2003	DQ194871
RV1505	2514/08/03	Namibia	Etosha National Park	Jackal	2003	DQ194872
RV1506	2627/08/03	Namibia	Etosha National Park	Jackal	2003	DQ194873
RV1507	2643/08/03	Namibia	Etosha National Park	Jackal	2003	DQ194874
RV1508	2702/09/03	Namibia	Windhoek	Jackal	2003	DQ194875
RV1509	2834/09/03	Namibia	Grootfontein	Jackal	2003	DQ194876
RV1510	2279/09/00	Namibia	Oshikoto	Dog	2000	DQ194877
RV1511	2329/10/00	Namibia	Oshakati	Dog	2000	DQ194878
RV1513	1711/06/03	Namibia	Grootfontein	Dog	2003	DQ194879
RV1514	1714/06/03	Namibia	Rundu	Dog	2003	DQ194880
RV1516	2001/06/03	Namibia	Grootfontein	Dog	2003	DQ194881
RV1517	2175/06/03	Namibia	Okahandja	Dog	2003	DQ194882
RV1518	3259/10/03	Namibia	Omaruru	Eland	2003	DQ194883
RV1519	2515/08/03	Namibia	Etosha	Hyena	2003	DQ194884
RV1826	NBA2	Namibia	Etosha	Bat-eared fox	NK	DQ194885
RV1827	NBA3	Namibia	Etosha	Bat-eared fox	NK	DQ194886
RV1829	NBA5	Namibia	Etosha	Jackal	NK	DQ194887
RV1830	NBA6	Namibia	NK	Dog	NK	DQ194888
RV1843	2693/80	Namibia	Windhoek	Kudu	1980	DQ194889
RV1875	993/80	Namibia	Windhoek	Dog	1980	DQ194890
RV1917	SK0006	Namibia	Keetmanshoop	Mongoose	NK	DQ194891
8708NAM	8708NAM	Namibia	NK	Kudu	1987	U22632
9227NAM	9227NAM	Namibia	NK	Jackal	1992	U22649
9365NAM	9365NAM	Namibia	NK	Human	1983	U22859
RV996	643/00	South Africa	Namaqualand	Aardwolf	2000	DQ194892
RV1005	836/00	South Africa	N. Cape	Genet	2000	DQ194893
RV1010	897/00	South Africa	N. Cape	Meercat	2000	DQ194894
RV1817	20/90	South Africa	Kernhardt	Bat-eared fox	1990	DQ194895
RV1841	1070/80	South Africa	Neudorf	Jackal	1980	DQ194900
RV1929	168/01	South Africa	N. Cape	Bat-eared fox	2001	DQ194901
RV1933	756/02	South Africa	N. Cape	Bat-eared fox	2002	DQ194902
RV135	947/80	South Africa	NK	Kudu	1980	DQ194903
2900AFS	2900AFS	South Africa	NK	Water mongoose	1991	U22861
8721AFS	8721AFS	South Africa	NK	Human	1981	U22633
9362AFS	9362AFS	South Africa	NK	Human	1988	U22860
9363ZAM	9363ZAM	Zambia	NK	Human	1991	U22857

Key: NK: not known

## References

[B1] Swanepoel R, Barnard BJH, Meredith CD, Bishop GC, Brückner GK, Foggin CM, Hübschle OJB (1993). Rabies in southern Africa. Onderstepoort J Vet Res.

[B2] King AA, Meredith CD, Thomson GR (1993). Canid and viverrid rabies viruses in South Africa. Onderstepoort J Vet Res.

[B3] von Teichman BF, Thomson GR, Meredith CD, Nel LH (1995). Molecular epidemiology of rabies virus in South Africa: evidence for two distinct virus groups. J Gen Virol.

[B4] Johnson N, Letshwenyo M, Baipoledi EK, Thobokwe G, Fooks AR (2004). Molecular epidemiology of rabies in Botswana: a comparison between antibody typing and nucleotide sequence phylogeny. Vet Microbiol.

[B5] Nel LH, Sabeta CT, von Teichman B, Jaftha JB, Rupprecht CE, Bingham J (2005). Mongoose rabies in southern Africa: a re-evaluation based on molecular epidemiology. Virus Research.

[B6] Courtin F, Carpenter TE, Paskin RD, Chomel BB (2000). Temporal patterns of domestic and wildlife rabies in central Namibia stock-ranching area, 1986–1996. Prev Vet Med.

[B7] Schneider HP, Kuwert, Merieux, Koprowski, Bogel (1985). Rabies in South Western Africa/Namibia. Rabies in the Tropics.

[B8] Hübschle OJB (1988). Rabies in the kudu antelope (*Tragelaphus Strepsiceros*). Rev of Infect Dis.

[B9] Snyman PS (1940). The study and control of the vectors of rabies in South Africa. Onderstepoort J Vet Sci and Anim Husb.

[B10] Meredith CD (1982). Wildlife rabies: past and present in South Africa. Sth Afr J Sci.

[B11] von Maltitz L (1950). Rabies in the northern districts of South West Africa. J Sth Afr Vet Med Assoc.

[B12] Alexander RA (1952). Rabies in South Africa: a review of the present position. J Sth Afr Med Assoc.

[B13] Sabeta CT, Bingham J, Nel LH (2003). Molecular epidemiology of canid rabies in Zimbabwe and South Africa. Virus Research.

[B14] Nadin-Davis SA, Bingham J, King AA, Fooks AR, Aubert M, Wanderler AI (2004). Europe as a source of rabies for the rest of the world – chapter 19. Historical perspective of Rabies in Europe and the Mediterranean Basin.

[B15] Barnard BJH, Hassel RH (1981). Rabies in kudus (*Tragelaphus Strepsiceros*) in South West Africa/Namibia. J South Afr Vet Assoc.

[B16] Berry HH (1993). Surveillance and control of anthrax and rabies in wild herbivores and carnivores in Namibia. Rev Sci tech Off int Epiz.

[B17] Office Internationale des Epizooties : HANDISTATUS II Namibia/rabies multiannual animal disease status. http://www.oie.int/hs2.

[B18] Barnard BJH, Hassel RH, Geyer HJ, de Koker WC (1982). Non-bite transmission of rabies in kudu (*Tragelaphus Strepsiceros*). Onderstepoort J Vet Res.

[B19] Johnson N, McElhinney LM, Smith J, Lowings P, Fooks AR (2002). Phylogenetic comparison of the genus Lyssavirus using distal coding sequences of the glycoprotein and nucleoprotein genes. Arch Virol.

[B20] Kissi B, Tordo N, Bourhy H (1995). Genetic polymorphism in the rabies virus nucleoprotein gene. Virology.

[B21] Barnard BJH (1979). The role played by wildlife in the epizootiology of rabies in South Africa and south-west Africa. Onderstepoort J Vet Res.

[B22] Chare ER, Gould EA, Holmes EC (2003). Phylogenetic analysis reveals a low rate of homologous recombination in negative-sense RNA viruses. J Gen Virol.

[B23] Hassel RH (1982). Incidence of Rabies in Kudu in South West Africa/Namibia. Sth Afr J Sci.

[B24] Nel JAJ (1993). The bat-eared fox: a prime candidate for rabies vector?. Onderstepoort J Vet Res.

[B25] Shaw JJH (1980). Rabies in Kudu [letter to the Editor; in Afrikaans]. J South Afr Vet Assoc.

[B26] Heaton PR, Johnstone P, McElhinney LM, Cowley R, O'Sullivan E, Whitby JE (1997). Heminested PCR assay detection of six genotypes of rabies and rabies-related viruses. J Clin Microbiol.

[B27] Thompson JD, Higgins DG, Gibson T (1994). CLUSTAL W : improving the sensitivity of progressive multiple sequence alignment through sequence weighting, position-specific gap penalties and weight matrix choice. Nucleic Acids Res.

[B28] Strimmer K, von Haeseler A (1996). Quartet puzzling: a quartet maximum likelihood method for reconstructing tree topologies. Mol Biol Evol.

[B29] Felsenstein J (1989). PHYLIP- Phylogeny Inference Package Version 3.2. Cladistics.

[B30] Page R (1996). Treeview: An application to display phylogenetic trees on personal computers. Comput Appl Biosci.

